# Collective interests, health research ethics and data governance for Indigenous Sámi populations

**DOI:** 10.1371/journal.pmed.1005250

**Published:** 2026-09-14

**Authors:** Susanna Ragnhild Andersdatter Siri, Christina Storm Mienna, Per Axelsson

**Affiliations:** 1 Centre for Sami Health Research, Department of Community Medicine, Faculty of Health Sciences, UiT The Arctic University of Norway, Tromsø, Norway; 2 Várdduo- Centre for Sámi Research, Faculty of Arts and Humanities, Umeå University, Umeå, Sweden; 3 Department of Odontology, Faculty of Medicine, Umeå University, Umeå, Sweden; 4 Department of Historical, Philosophical and Religious Studies, Faculty of Arts and Humanities, Umeå University, Umeå, Sweden

## Abstract

In this Perspective, Susanna Siri and colleagues examine ethical guidelines and governance principles relevant to the collection, use, reuse and storage of data for the Sámi population and Indigenous peoples, and provide recommendations on best practice.

## Teaser

The General Data Protection Regulation (GDPR) provides a robust framework for regulating individual data collection, use, reuse, and storage across Europe, but offers limited safeguards for collective rights, such as those of Indigenous peoples. Here, we examine specific ethical guidelines and governance principles relevant to the Sámi population, and those general for Indigenous peoples, and offer recommendations for best practices in health research.

## Introduction

The General Data Protection Regulation (GDPR) provides limited safeguards for collective rights, such as those of Indigenous peoples (IP). Open science practices, guided by the Findable, Accessible, Interoperable, Reusable (FAIR) principles, aim to enhance data transparency and facilitate data reuse [1]. However, these principles may violate IP rights, as Indigenous self-determination includes the authority to govern Indigenous data, limiting its openness and reuse. In response, the Collective Benefit, Authority to Control, Responsibility, and Ethics (CARE) principles were developed to complement the FAIR principles by emphasising that data is embedded within the social, cultural and legal contexts of the peoples from whom it originates [2]. Importantly, the CARE principles encourage the application of local IP ethical and data governance protocols [3], addressing collective rights not explicitly covered by the GDPR. This Perspective examines how existing Sámi health research guidelines align with the CARE principles.

The United Nations Declaration on the Rights of Indigenous Peoples (UNDRIP) recognises IP’s rights to self-determination and to maintain and strengthen their own institutions and decision-making. The Sámi are the IP of Sápmi, a region spanning northern Norway, Sweden, Finland, and the Kola Peninsula in Russia with distinct languages, traditions, and cultural practices [4]. The country-specific Sámi Parliaments in Norway, Sweden and Finland are the highest governing bodies for Sámi people. In Norway [5] and Finland [6], Truth and Reconciliation Commissions, and in Sweden a Truth Commission [7], document the historical harms caused by assimilation policies and propose measures to strengthen Sámi self-determination. Against this backdrop, an important question is how Sámi collective interests are governed within health research.

## Navigating differing requirements across Norway, Sweden and Finland

In Norway, collection or use of Sámi data for all health research (except use of health or social care services) requires individual and collective Free Prior Informed Consent (FPIC). Collective consent is requested when the research includes the Sámi people as a group, or includes places/regions where the Sámi constitute a significant proportion of the population [8]. In 2020, the Norwegian Sámi Parliament appointed an Ethical Committee for Sámi Health Research to review research applications and provide collective consent. The Committee seeks to ensure that research projects are conducted in a culturally safe manner, in partnership with Sámi communities and participants. The guidelines are grounded in Sámi core values, including respect, responsibility, partnership, self-determination, and with the intention of collective benefits for present and future generations. They also state that Sámi participants have the right to control and access research data, i.e., the management of data in registries, biobanks and the direct use of results from Sámi research, which must be described in the collective consent application [8].

Sweden lacks Sámi ethical research guidelines for health and other research disciplines. However, in 2024, the Swedish Research Council incorporated the CARE principles into its Good Research Practice, thereby acknowledging—but not providing concrete guidance on—how research projects can uphold Sámi collective rights to data. Sámiiid Riikkasearvi (SSR), an interest organisation representing Sámi who are members of a reindeer herding community or a Sámi association in Sweden, published in 2019 a policy on how to conduct research with SSR [9]. The policy states that an individual and collective FPIC is mandatory and should be formalised with an agreement addressing current and future data management. Notably, the SSR offers a preparatory course for researchers to ensure cultural sensitivity [9]. Due to the absence of national Sámi ethical research guidelines, researchers have increasingly adopted the SSR guidelines, in addition to the Norwegian Sámi ethical guidelines [8].

The Sámi Ethical guidelines for research in Finland from 2024 are multidisciplinary and applicable across Sápmi if local Sámi research guidelines are missing [10]. The Finnish guidelines address how to conduct ethical research with Sámi communities and also provide concrete actions to improve Sámi peoples’ rights to control their data. Specifically, the importance of agreeing and developing a plan with the Sámi community/participants on what type of data is collected, how data is processed, used, reused, and stored, determining ownership, and whether data can be shared and is accessible to the community/participants. In Finland, researchers are expected to comply with the Sámi ethical guidelines through self-regulation, as there is no review board to oversee their implementation. Moreover, if research data that includes collective immaterial rights is used for commercialisation or patenting, an explicit agreement is required with rightsholders, together with fair compensation [10], similar to what is demanded by the Norwegian Sámi health research guidelines [8].

The SAMINOR study is a population-based survey on health and living conditions in regions with Sámi and non- Sámi populations and exemplifies how Sámi data governance can be exercised [11]. The university is the data controller, with data infrastructure and data management units. SAMINOR data are accessible from open platforms, together with metadata describing the data’s sensitive nature and that a Sámi collective consent is required. Applications are evaluated by the SAMINOR project board. Before data is handed out, the board ensures that: applicants possess knowledge of Sámi culture, language, and society; that collective consent is obtained; that the project fulfils the overall aim of the SAMINOR Study; and is approved by an ethical review board. If these are fulfilled, a time-restricted contract is written between the data processing institution, i.e., the applicants’ hosting institution and data controller. A dynamic Data Protection Investigation Assessment plan describing mitigation of re-identification and anonymisation is mandatory when handling sensitive individual data. Universities and funders may require data to comply with the FAIR principles, hence, a Data Management Plan (DMP) is written describing how data is managed, secured, stored, and shared with researchers nationally or abroad—which demands a Joint Controller Agreement (JCA) or Data Processing Agreement (DPA) depending on the level of collaboration.

## Applying SODA and CARE principles

At a transnational level, Sápmi has ethical and governing principles, known as the Sámi Ownership and Data Access (SODA) principles, adopted by the Saami Council in 2024 [12]. The Saami Council is a cross-border Sámi representative non-governmental organisation that promotes Sámi interests and rights. The SODA principles are fundamental for any collaborations with the Saami Council and may also be applied by Sámi institutions contributing to data collections. Building on the CARE principles, SODA seeks to support Sámi dignity, wellbeing, and autonomy by strengthening Sámi ownership, control and access to data. As ownership is included, the SODA principles resemble the well-known Ownership, Control, Access and Possession principles that have existed since 1998 for First Nations in Canada. Universities are often data controllers under GDPR and assert ownership of research data, which may conflict with the principle of ownership as articulated by the SODA principles.

The CARE principles address the historical and ongoing injustices IP face as a result of colonisation and are interrelated and mutually reinforcing. Even if the Sámi Ethical guidelines’ primary focus is “Ethics”, they address data governance, particularly the Finnish [10], SSR guidelines [9] and the SODA principles, in ways that make researchers accountable for addressing Sámi needs and interests in data. For instance, the use of Sámi languages is emphasised in all Sámi ethical guidelines and should be used whenever possible, in line with participants’ needs, across all stages of the research process, including data collection, analysis, dissemination, and scientific writing [8–10]. Notably, the Finnish ethical guidelines explicitly address capacity-building by strengthening Sámi communities and supporting Sámi data governance through increased data accessibility. In terms of “Collective Benefits”, the guidelines in Norway, Finland and SSR in Sweden require engagement and equal partnership with Sámi communities/participants, alongside a risk assessment of the impacts of the research, including harms and benefits for future generations of Sámi people [8–10]. The “Authority to Control” principle is most explicitly stated in the Finnish [10] and SODA guidelines [11]. The Finnish guidelines recommends storing Sámi data in Sámi museums, institutions or archives, together with a written agreement on data collection, processing, reuse, storage, ownership, and sharing—agreed upon with the Sámi research partner(s). With the exception of the SODA principles, Sámi ethical guidelines do not explicitly address “Responsibility”. However, all the ethical guidelines require the researchers to possess knowledge of Sámi health [8], reindeer herding [9], values, traditions, history, traditional knowledge, language, social relations, and culture before initiating engagement, which is needed to promote responsible research and to create and maintain a culturally safe research environment [8–10].

## Recommendations for best practice

Recent work has demonstrated how the CARE principles can be translated into concrete practices for data repositories and stewardship [13]. Currently, there is no formal mechanism within institutions or ethical review processes in Sápmi for securing that a collective consent is given for research involving Sámi people, except for the ethics review board in northern Norway. Whether such consent is sought therefore remains largely at the discretion of individual researchers. To support responsible governance of Sámi research data, Table 1 integrates the CARE Principles and Sámi ethical guidelines and considers their alignment with the GDPR.

**Table 1:** Key recommendations for addressing CARE principles and Sámi ethical guidelines, with suggestions on how to address these within the GDPR framework.

Principles	Recommendations	How to?	GDPR alignments
**Collective benefit**	**Design research to generate benefits**	Discuss with Sámi partners what they expect and need. Consider benefits, harms, and impacts on future generations. Communicate the results back to the communities in their language. Store data in their institutions, and in formats that are accessible.	All data processing should have a specified purpose, which participants are informed about
	**Engage Sámi stakeholders**	Establish partnership with Sámi communities, organisations, institutions, or representative bodies early during project planning, and maintain partnership throughout the project.	
**Authority to Control**	**Respect individual and collective interests**	Obtain Free Prior Informed Consent (FPIC) and consider collective consent, consultation, or community approvals is required*	
	**Develop a data governance plan**	Agree with participants/communities on what data will be collected, who can access it, how it is used and reused, shared, archived or transferred between institutions. Include Sámi in governance structures, for example, establish a Sámi advisory board, Sámi data access committees, and Sámi ethics review.	Consider writing a Joint Controller Agreement (JCA) between Sámi communities and the institution, or Data Processing Agreements (DPA) with researchers
	**Incorporate Indigenous governance mechanisms**	Use collective consent requirements and include Sámi in governance structures when evaluating requests for data access, use and reuse.	Same as above.
**Responsibility**	**Ensure researchers are culturally competent**	Researchers should demonstrate knowledge (to work respectfully) of Sámi history, culture, language, health, traditional knowledge, and social conditions *before* initiating projects.	
	**Include CARE considerations**	Include Sámi governance and potential licenses (agreed upon with Sámi participants) and apply these into DMP	Document data stewardship, storage, security, sharing, and access in DMP
**Ethics**	**Align FAIR and CARE**	Metadata available while access to sensitive data is restricted or requires collective approvals [13].	
	**Ensure accountability and transparency**	Clearly document consent procedures, governance decisions, restrictions on use, and conditions for reuse.	
	**Address benefit-sharing**	Commercialisation, patenting, or use of collective cultural and intellectual resources should be based on explicit agreements with relevant Sámi rights holders	

Examine CARE principles in relation to the CARE maturity model, accessible from: https://indigenousdatalab.org/care-data-maturity-model.

*Conditions for collective consent within Sámi health research in Norway, Finland and Sweden [8–10]. Note the legal obligations beyond Sámi collective interest.

Abbreviations: CARE, Collective Benefit, Authority to Control, Responsibility, and Ethics; General Data Protection Regulation, GDPR.

## Abbreviations

DMP: Data Management Plans
DPA: Data Processing Agreements
FAIR: Findable, Accessible, Interoperable, Reusable
FPIC: Free Prior Informed Consent
GDPR: General Data Protection Regulation
IP: Indigenous peoples
JCA: Joint Controller Agreement
SODA: Sámi Ownership and Data Access
SSR: Sámiiid Riikkasearvi
UNDRIP: United Nations Declaration on the Rights of Indigenous Peoples
